# Microneedles loaded with glutathione‐scavenging composites for nitric oxide enhanced photodynamic therapy of melanoma

**DOI:** 10.1002/btm2.10352

**Published:** 2022-06-17

**Authors:** Fan Jia, Weijiang Yu, Xinfang Li, Yonghang Chen, Youxiang Wang, Jian Ji

**Affiliations:** ^1^ MOE Key Laboratory of Macromolecule Synthesis and Functionalization of Ministry of Education, Department of Polymer Science and Engineering Zhejiang University Hangzhou Zhejiang China

**Keywords:** melanoma, microneedles, nanocomposites, nitric oxide, photodynamic therapy

## Abstract

Photodynamic therapy (PDT) represents an attractive promising route for melanoma treatment. However, its therapeutic efficacy is compromised by inefficient drug delivery and high glutathione (GSH) levels in cancer cells. To overcome these challenges, microneedles (MNs) system loaded with GSH‐scavenging nanocomposites was presented for nitric oxide (NO) enhanced PDT. The nanocomposites consisted of *S*‐nitroso‐*N*‐acrylate penicillamine (SNAP; a NO donor) grafted fourth‐generation polyamide amine dendrimer (G_4_) and chlorin e6 (Ce6). Upon local insertion of polyvinylpyrrolidone MNs, G_4_‐SNAP/Ce6 composites were fast delivered and significantly amplified the therapeutic effects during PDT, via GSH depletion and reactive nitrogen species generation. Even with a single administration and low power light exposure, MNs with G_4_‐SNAP/Ce6 effectively halt the tumor progression. The system demonstrated better cancer ablation efficacy than Ce6 alone toward melanoma. The strategy may inspire new ideas for future PDT‐related therapy for skin tumors.

## 1 INTRODUCTION

Melanoma is one of the most aggressive cancers in humans, especially in aging people. The rapid progression contributes to its malignancy, causing a rather unsatisfied 5‐year survival rate in clinics.^1^, ^2^, ^3^ Many treatments are developed to fight against melanoma cancer. Among them, photodynamic therapy (PDT) has attracted rising attention both in research and clinical applications.^4^, ^5^, ^6^, ^7^ During standard PDT, photosensitizers (PS) are intravenously administrated and subsequently activated by light in tumor sites to produce tumor‐destroying reactive oxygen species (ROS).^8^, ^9^, ^10^ However, systemic administration of PS leads to limited drug enrichment in targeted lesions of tumors.^11^ It unavoidably brings about an accumulation of PS in healthy superficial organs or tissues as a side effect.^12^, ^13^ When exposed to daylight, the generated ROS damages the DNA of normal skin cells and ironically promotes the potential occurrence of skin cancer. Besides, tumor cells produce a high level of intracellular glutathione (GSH).^14^, ^15^, ^16^ As a naturally occurring reductant, GSH is a potent scavenger of various oxidants. It protects cancer cells from ablation of ROS, thus dampening the outcome of PDT. There is always a long‐lasting need to exploit new material strategies with higher delivering precision and GSH‐scavenging ability to enhance PDT.

Microneedles (MNs) have emerged as an innovative platform for efficient transdermal drug delivery in recent years.^17^, ^18^, ^19^ They are quite flexible in cargo selection, facile in manufacture, and minimally invasive to the patients.^20^, ^21^, ^22^, ^23^, ^24^, ^25^ More importantly, local administration gives MNs a peerless edge in the accuracy of drug delivery, making it an ideal method to treat melanoma tumors and other cutaneous illnesses.^26^, ^27^, ^28^, ^29^, ^30^ It is an excellent choice to realize targeted delivery of PS and to avoid the side effects of PDT.

On the other hand, *S*‐nitrosothiols (RSNOs) are a large family of compounds, many of which are found in the human body as native carriers for nitric oxide (NO) over a long distance.^31^, ^32^, ^33^ They are generally considered to have good safety and can release NO by consuming a high level of GSH inside cells.^34^, ^35^, ^36^, ^37^ Interestingly, NO can reduce intracellular GSH levels via various bio‐metabolism as well.^38^, ^39^, ^40^ It can also react with ROS to produce peroxynitrite anions (ONOO^−^) or other reactive nitrogen species (RNS), which are more lethal than either ROS or NO.^41^, ^42^ Such benefits make RSNOs a promising choice for PDT to resist consumption by GSH and boost efficiency.

**SCHEME 1:**
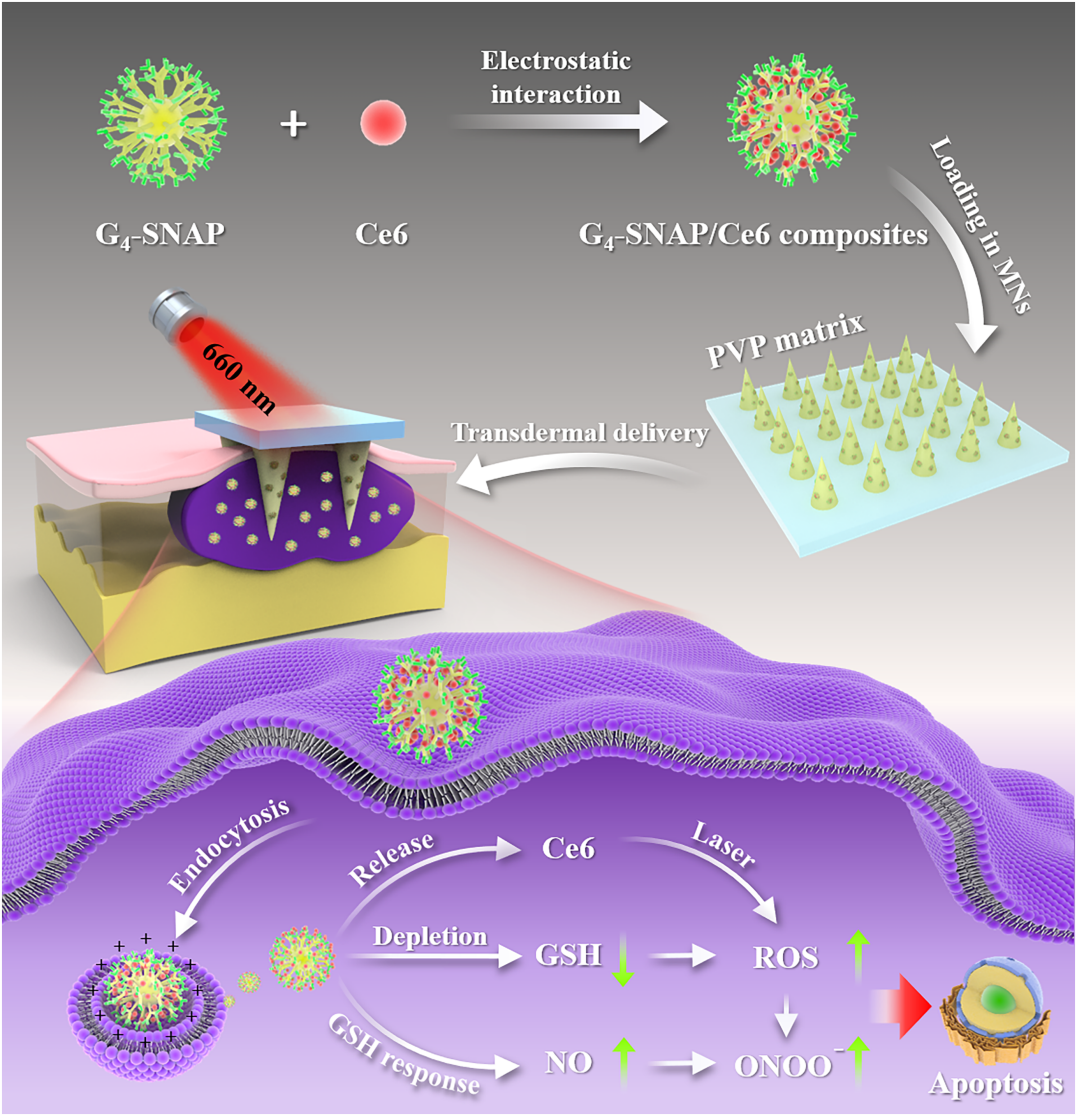
Microneedles loaded with GSH consuming RNS generators for enhanced photodynamic therapy of melanoma. GSH, glutathione; RNS, reactive nitrogen species

Hence, in the present study, we combined NO gas therapy and MNs to overcome the drawbacks of PDT mentioned above. GSH responsive NO donor, *S*‐nitroso‐*N*‐acrylate penicillamine (SNAP), was conjugated onto the outer amine groups of fourth‐generation polyamidoamine dendrimer (G_4_‐NH_2_) to yield a GSH consuming scaffold. The positive charges of the SNAP modified dendrimer could attract and pack negatively charged chlorine 6 (Ce6) inside via gentle procedure, without compromising the attached NO. Since NO is highly susceptible to scavenging, such a co‐loading method is beneficial to maximizing synergistic effects between NO and PDT. The Ce6/NO co‐loaded dendrimer was then encapsulated in dissolving MNs, using biocompatible polyvinylpyrrolidone (PVP) as the matrix. We hypothesized that the SNAP and subsequently produced NO could deplete the high‐level GSH inside melanoma tumor cells upon topical inoculation of MNs, thus improving the therapeutic outcome of the following PDT (Scheme 1). The present work might provide new inspiration for future skin cancer therapy.

## 2 EXPERIMENTAL SECTION

### 2.1 Materials

Poly(amidoamine) dendrimer (generation 4.0, G_4_‐NH_2_, water solution, 10% wt/wt) was purchased from Dendritech. *tert*‐Butyl nitrite (TBN) and fluorescein 5(6)‐isothiocyanate (FITC, ≥90%) was obtained from Sigma‐Aldrich. PVP (average Mw = 40 kDa), *N*‐acetyl‐d‐penicillamine (NAP), and chlorin e6 (Ce6) were purchased from Aladdin. All other chemicals and solvents were used without further purification unless mentioned. Cell Counting Kit‐8 (CCK‐8) was obtained from Dojindo Molecular Technologies. Polydimethylsiloxane (PDMS) female molds (height: ~1000 μm, base width: ~500 μm, spacing: ~1000 μm, 5 × 5 array) were purchased from the Research Institute of Zhejiang University‐Taizhou. Optimum cutting temperature (OCT) compounds were provided by Sakura Finetek.

### 2.2 Synthesis and characterization of G_4_‐NAP, G_4_‐SNAP(‐FITC), and G_4_‐SNAP(‐FITC)/Ce6

G_4_‐NAP was synthesized through a multi‐step synthetic route. First, NAP thiolactone (NAP‐TL) was synthesized according to previous research. In brief, NAP (3.0 g) was suspended into pyridine (10.0 ml) and kept at 0°C for 0.5 h. Then a mixture of 20 ml of pyridine/acetic anhydride (vol:vol = 1:1) solution was added dropwise. After stirring overnight, the solvents were removed under reduced pressure. The obtained solid was dissolved in chloroform and extracted with 1 M hydrochloric acid three times. The chloroform layer was dewatered over sodium sulfate and filtered. The crystalline product of NAP‐TL was obtained by rotating evaporation and recrystallized from hexanes (2.0 g; 73.5%; CDCl_3_ for ^1^H NMR). Second, G_4_‐NH_2_ (0.20 g) and NAP‐TL (0.33 g) were added into dimethylformamide (DMF; 5 ml) and then stirred at room temperature for 24 h. The obtained solution was dropped into excessive diethyl ether and the precipitant was centrifuged. The obtained solid was dissolved in distilled water and dialyzed overnight (Mw = 5 kDa). The solid was obtained via frozen drying (0.5 g; 95%; D_2_O for ^1^H NMR).

G_4_‐SNAP was synthesized by TBN and G_4_‐NAP. Typically, TBN (1.5 ml) and G_4_‐NAP (0.2 g) were added into DMF (5 ml) at 0°C. After stirring in the dark overnight, the final solution was precipitated in diethyl ether and the solid was dissolved again and dialyzed (Mw = 5 kDa) against distilled water for 24 h and then frozen dried (0.2 g; >95%; D_2_O for ^1^H NMR).

To obtain G_4_‐SNAP‐FITC, excessive FITC (30 mg) and G_4_‐SNAP (100 mg) were dissolved in water and stirred in the dark overnight. After that, the mixture was dialyzed against distilled water (Mw = 5 kDa) and then frozen dried (0.1 g; >95%; D_2_O for ^1^H NMR).

To obtain G_4_‐SNAP(‐FITC)/Ce6, G_4_‐SNAP(‐FITC) (200 mg), and Ce6 (20 mg) were mixed in the DMF/water solution (vol:vol = 1:1) overnight. The final solution was dialyzed against distilled water (Mw = 5 kDa) for 24 h and then frozen dried.

^1^H NMR spectra were obtained from an NMR spectrometer (DMX 500; Bruker). Morphology, particle size distribution, and zeta potential of nanoparticles were obtained on transmission electron microscopy (HT7700; Hitachi) and Zetasizer Nano‐ZS (Malvern). UV–vis spectra were recorded on a UV–vis spectrometer (UV‐2550; Shimadzu).

### 2.3 Cell culture

Human melanoma A375 cells were purchased from Tong Pai (Shanghai) Biotechnology Co. LTD. The cells were cultured in Dulbecco's modified Eagle's medium (DMEM) containing 1% (vol/vol) penicillin–streptomycin and 10% (vol/vol) fetal bovine serum under a humidified atmosphere (90%) containing CO_2_ (5%) at 37°C.

### 2.4 Cellular uptake studies

A375 cells were seeded into 35‐mm glass‐bottom cell culture dishes at a density of 2 × 10^5^ cells in 1 ml DMEM medium per dish. After 24‐h incubation, cells were treated with G_4_‐SNAP‐FITC/Ce6 composites (Ce6 10 μg/ml and NO 10 μg/ml) for 2 h. After washing dishes with phosphate‐buffered saline (PBS) three times, these cells were fixed with a 4% paraformaldehyde solution for 20 min. The cell nuclei were stained with DAPI for 20 min and washed with PBS three times before imaging by confocal microscopy (LSM780; ZEISS).

### 2.5 GSH measurement

Cellular GSH levels were determined by the mercapto assay kit (BC1370; Solarbio). The thiol groups on GSH could react with 5,5′‐dithiobis‐(2‐nitrobenzoic acid) and generate a yellow compound that had maximum absorption at 412 nm. To study the GSH depletion ability of G_4_‐SNAP/Ce6, different volumes of G_4_‐SNAP/Ce6 solutions were directly added into GSH solutions with final NO/GSH molar concentration ratios at 0, 0.2, 0.5, and 1, respectively. To study the intracellular GSH levels, A375 cells were seeded into 24‐well plates at a density of 10^5^ cells per well. After incubating with G_4_/Ce6 (Ce6 10 μg/ml), G_4_‐SNAP (NO 10 μg/ml), G_4_‐SNAP/Ce6 (Ce6 10 μg/ml and NO 10 μg/ml) for 3 h, the cells were washed with PBS and lysed by repeated freeze–thaw. The GSH levels of samples were measured by the absorbance at 412 nm using a UV–vis spectrometer (UV‐2550; Shimadzu).

For tumors, excised tissues were quenched in liquid nitrogen and subjected to freeze‐drying. 10 mg of dry tissues were homogenated into suspension in 100‐μl PBS and centrifuged. The supernate was collected and protein levels were determined to standardize the number of samples before the test. The GSH level was measured similarly to the mentioned above. For total GSH concentration, samples were first incubated with NADPH, before being measured.

### 2.6 GSH triggered NO release

To investigate GSH triggered NO release from G_4_‐SNAP/Ce6, 20 μl of PBS with different GSH concentrations were added to 1.98 ml G_4_‐SNAP/Ce6 solution (NO 100 μg/ml), with a final GSH concentration of 0 μM, 2 μM, and 5 mM, respectively. The NO release profiles were recorded by Free Radical Analyzer (TBR 4100; WPI) equipped with a NO detector.

### 2.7 Intracellular Ce6, NO detection

A375 cells were seeded into 24‐well plates at a density of 2 × 10^4^ cells in 500 μl DMEM medium per well. DAF‐FM DA (S0025; Beyotime) was used to test the intracellular NO generation. DAF‐FM DA solution was added to A375 cells for 20‐min incubation and then removed. PBS, free Ce6 (10 μg/ml), G_4_‐SNAP/Ce6 (NO 10 μg/ml and Ce6 10 μg/ml), or G_4_‐SNAP/Ce6 (Ce6 10 μg/ml, incubating at 60°C for 4 h to release NO) was added for another 3‐h incubation, respectively. After washing with PBS, cell imaging was performed using a fluorescence microscope (IX81; Olympus) (NO: *λ*_ex_ = 488 nm; Ce6: *λ*_ex_ = 565 nm).

### 2.8 ROS generation and ONOO^−^ detection

DCFH‐DA (S0033; Beyotime) was used to investigate intracellular ROS generation. Typically, A375 cells were seeded into 24‐well plates at a density of 2 × 10^4^ cells in 500 μl DMEM medium per well. After treating with PBS, free Ce6 (10 μg/ml), G_4_‐SNAP/Ce6 (Ce6 10 μg/ml and NO 10 μg/ml), G_4_‐SNAP/Ce6 (Ce6 10 μg/ml, incubating at 60°C for 4 h to release NO) for 3 h, A375 cells were cultured in 200 μl diluted DCFH‐DA solution (1:1000) for 20 min. After washing with PBS, cells were exposed to 660 nm light (0.20 W/cm^2^, 1 min) and observed under a fluorescence microscope (IX81; Olympus).

ONOO^−^ fluorescent probe (BB‐470568; BestBio) was used to investigate the intracellular peroxynitrite anions (ONOO^−^) generation. Typically, A375 cells were seeded into 24‐well plates at a density of 2 × 10^4^ cells in 500 μl DMEM medium per well. After treating with PBS, free Ce6 (Ce6 10 μg/ml), G_4_‐SNAP (NO 10 μg/ml), G_4_‐SNAP/Ce6 (Ce6 10 μg/ml and NO 10 μg/ml) for 3 h, A375 cells were cultured in 200 μl diluted ONOO^−^ fluorescent probe solution (1:1000) for 20 min. After washing with PBS, cells were exposed to 660 nm light (0.20 W/cm^2^, 1 min) and observed under a fluorescence microscope (IX81; Olympus).

For tumors, 50‐mg excised tissues were homogenated into suspension in 500 μl PBS and centrifuged. The supernates were collected and protein levels were determined to standardize the number of samples before the test. ROS levels were determined by an O12 fluorescent probe (maximum *λ*_ab_ = 488 nm, *λ*_em_ = 526 nm) on a SpectraMax M5/M5e microplate system (Molecμlar Devices). RNS was measured similarly with O52 as a probe (maximum *λ*_ab_ = 488 nm, *λ*_em_ = 530 nm).

### 2.9 Cytotoxicity assay

The cell cytotoxicity on A375 cells was performed by CCK‐8 assay. A375 cells were seeded into 96‐well plates at a density of 8000 cells in 200 μl DMEM medium per well. After 24 h incubation, cells were treated with different concentrations of Ce6, G_4_‐SNAP, and G_4_‐SNAP/Ce6, respectively, and incubated for another 3 h. After replacing with a fresh medium, cells were treated either with or without 660 nm light (0.2 W/cm^2^, 1 min) radiation and incubated for another 24 h. Then, CCK‐8 (20 μl/well) was added for another 4‐h incubation. Finally, OD450 was measured by a microplate reader (Thermo Fisher Scientific).

### 2.10 Fabrication and characterization of G_4_‐SNAP(‐FITC)/Ce6 loaded MNs

G_4_‐SNAP(‐FITC)/Ce6 loaded MNs were fabricated via a two‐casting method. In the first step, 5 mg G_4_‐SNAP(‐FITC)/Ce6 was mixed into a 0.3 ml casting solution containing 50% wt/vol PVP and then ~50 μl was applied to the MNs PDMS mold. After centrifugation (5000 rpm) for 10 min, the residue solution on the mold was removed by a spatula. These molds were allowed to dry further in a desiccator overnight. In the second step, PVP solution (100% wt/vol) was cast on the molds to form the backing of the MNs patch. After drying in the desiccator, these MNs patches were peeled off for further use. Morphology of MNs was acquired with a digital microscope and scanning electron microscope (SEM; S4800; Hitachi). A confocal microscope (LSM780; ZEISS) was employed to inspect the MNs loaded with G_4_‐SNAP‐FITC/Ce6. Three‐dimensional (3D) images of G_4_‐SNAP‐FITC and Ce6 distribution in MNs were reconstructed. The drug content of MNs was evaluated by UV–vis spectroscopy after dissolving in water. The drug delivery efficiency was determined by measuring the amount of G_4_‐SNAP/Ce6 composites lost from MNs. After being applied to the porcine skins at different times, the removal MNs were dissolved in water and the amount of G_4_‐SNAP/Ce6 composites was calculated according to the absorbance of Ce6 at 660 nm.

### 2.11 Skin penetration test

G_4_‐SNAP(‐FITC)/Ce6 loaded MNs patches were applied on porcine skins for 5 min. After removing MNs, skin samples were embedded in the OCT compound. Then, skin samples were cut into 12‐μm slices using a freezing microtome (CryoStar NX50; Thermo Fisher Scientific). These slices were stained with hematoxylin and eosin (H&E) staining for histological analysis. To observe the drug diffusion, the slices were observed by a fluorescence microscope (DS‐Ri2; Nikon).

### 2.12 Animals and tumor model

Male Balb/c nude mice (4‐week‐old) were purchased from the Zhejiang Academy of Medical Sciences. All animal experiments were performed strictly according to the “Principles of Laboratory Animal Care” (NIH publication no. 86‐23, revised 1985) and have received approval from the Lab Animal Welfare and Research Committee, Zhejiang University. The tumor volume was calculated as follows:

$$\text{tumor volume}=\frac{\text{tumor length}\times {\text{tumor width}}^{2}}{2}.$$

### 2.13 In vivo antitumor activity mediated by MNs

A375 cells (5 × 10^6^) were subcutaneously inoculated into the outer thigh of mice which induced one tumor at the local site. When the tumor volumes reached about 80–120 mm^3^, the mice were randomly divided into four groups (four per group). G_4_/Ce6 MNs (Ce6: 25 μg/patch), G_4_‐SNAP MNs (25 μg NO equivalent SNAP/patch), G_4_‐SNAP/Ce6 MNs (Ce6: 25 μg/patch; 25 μg NO equivalent SNAP/patch), or blank MNs were applied on the tumor and kept for 5 min, respectively. After removing MNs, these tumors were treated with 660 nm light irradiation (0.2 W/cm^2^, 1 min). Tumor volumes and body weights were recorded regularly during experiments. On Day 18, the tumors were resected for H&E, Ki‐67, or terminal deoxynucleotidyl transferase dUTP nick end labeling (TUNEL) staining.

### 2.14 Western blot assay

Western blot assay was used to detect the expression of nuclear factor erythroid 2‐related factor 2 (Nrf‐2), tumor necrosis factor‐α, cleaved caspase 3 and cleaved caspase 8 in melanoma tumor tissue after different treatments. The tissue was lysed and the protein was extracted. BCA Protein Quantification Kit was used to determine the total protein before the test. Then the proteins of each sample were separated by 10% sodium dodecyl sulfate–polyacrylamide gel electrophoresis (SDS‐PAGE). Then the PAGE was transferred onto the polyvinylidene fluoride membrane. The membrane was submerged in TBST containing 5% nonfat dry milk for 1 h and then incubated with relevant primary antibodies at 4°C overnight. After that, the membranes were washed three times with TBST and hybridized with relevant secondary antibodies at room temperature for 1 h. At last, the membranes were rinsed and visualized by chemiluminescence using an enhanced chemiluminescence detection kit. Protein expression was normalized to GADPH. The relative expression level was quantified with ImageJ software. The concentration of GSH, ROS, and RNS was quantified using similar methods to those in vitro experiments following standard protocols.

### 2.15 Statistics

All experiments were conducted with at least three replicates. All experimental data were presented as their mean ± *SD* and analyzed by one‐way ANOVA. *p < 0.05 was considered statistically significant.

## 3 RESULTS AND DISCUSSION

### 3.1 Synthesis and fabrication of SNAP/Ce6 co‐loaded dendrimer

**FIGURE 1:**
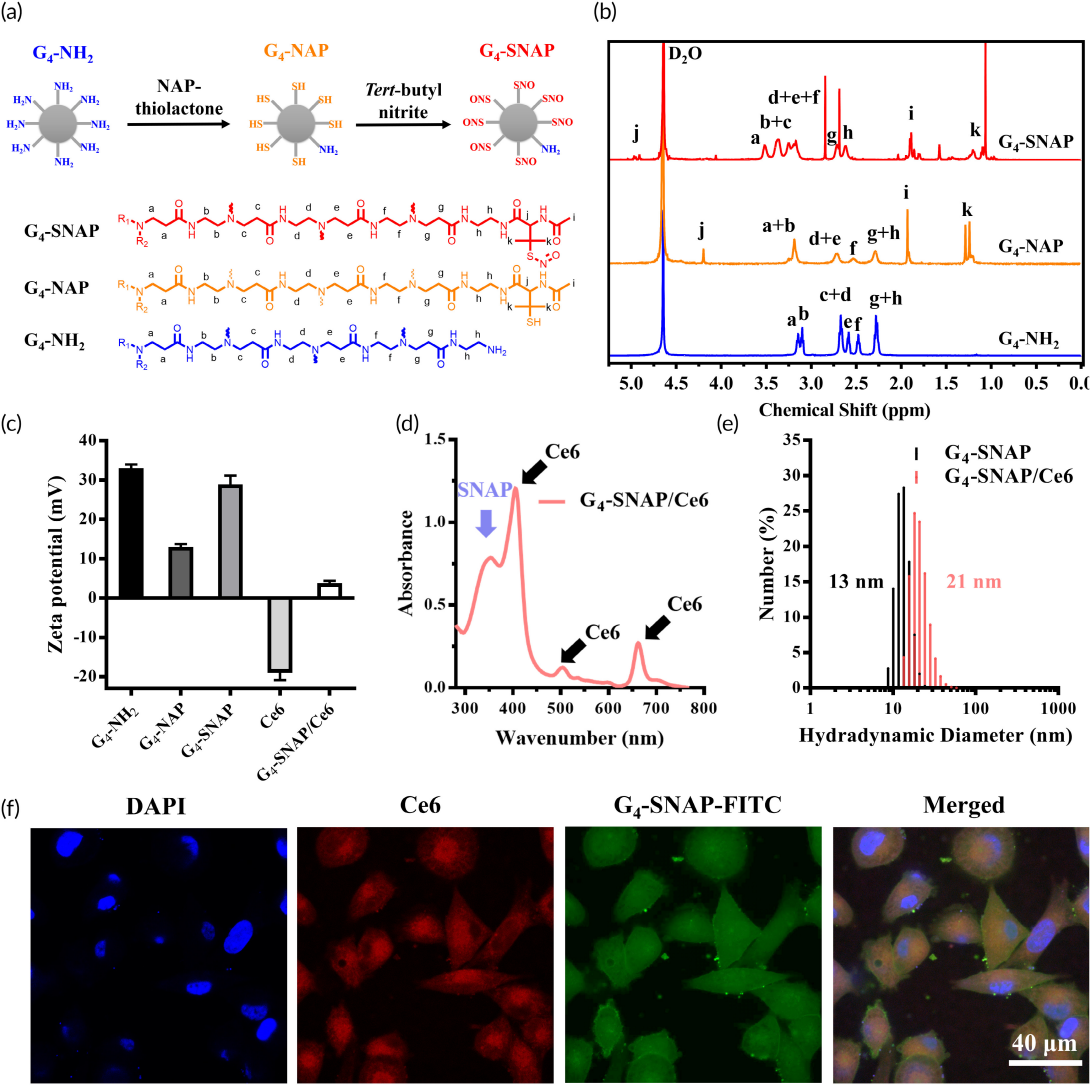
(a) Synthesis procedure illustration and representative chemical structures and (b) ^1^H NMR spectra of G_4_‐NH_2_ (blue line), G_4_‐NAP (yellow line), and G_4_‐SNAP (red line); (c) zeta potential of different dendrimers; (d) UV–Vis spectrum of G_4_‐SNAP/Ce6; (e) number average diameter of G_4_‐SNAP dendrimer before (black bars) and after (red bars) Ce6 encapsulation and (f) confocal microscopic images of A375 cells treated with FITC labeled G_4_‐SNAP/Ce6 (scale bar = 40 μm). Ce6, chlorin e6; NAP, *N*‐acetyl‐d‐penicillamine; NMR, nuclear magnetic resonance; SNAP, *S*‐nitroso‐*N*‐acrylate penicillamine

Different RSNOs have various stability. Their NO release half‐lives may range from a few seconds to tens of hours.^43^ The more stable the RSNOs are, the fewer NO losses before administration, hence the higher the delivering efficacy is. SNAP is one of the most stable RSNOs, due to unique molecular structures on its γ‐C.^44^, ^45^ The SNAP dendrimer (G_4_‐SNAP) was obtained via a three‐step process (Figures 1a and S1). The successful synthesis of each step was confirmed by ^1^H NMR results (Figures 1b and S2). The average grafting number on each dendrimer was calculated as 55 (conjugating ratio 86%). It was based on the ratio between the accumulated intensity of peaks at *δ* = 3.20 ppm and 1.25 ppm (Figure 1b), which belonged to methylenes of G_4_ dendrimer and methyl groups of SNAP, respectively. The zeta potential decreased from 30 to 4 mV after encapsulation of negatively charged Ce6 in the dendrimer (Figure 1c). UV–Vis spectrometry was further employed to monitor the formation of the G_4_‐SNAP/Ce6 composites. The peak at 343 nm in spectra corresponds to the characteristic absorbance of SNAP, and the peaks at 500, 660, and 400 nm correspond to the characteristic absorbance of Ce6 (as labeled in Figure 1d). The mass ratio of Ce6 in the G_4_‐SNAP/Ce6 composites was measured by UV–Vis spectrum at 660 nm and calculated as 6% according to a standard curve of Ce6 (Figure S3). The average hydrodynamic diameter of dendrimer increased from 13 to 21 nm, as indicated by DLS (Figure 1e). All these data suggested the successful loading of Ce6 in G_4_‐SNAP.

### 3.2 Endocytosis and colocalization of G_4_‐SNAP/Ce6 in A375 cells

A375 was one of the most malignant melanoma cancer cells. It was used as model cells for in vitro study in this work. To visualize the cellular uptake process, G_4_‐SNAP was labeled with FITC. The green fluorescent dye was chosen to distinguish it from the red fluorescence of Ce6. After 2 h of incubation with G_4_‐SNAP‐FITC/Ce6 composites, both bright red and green fluorescence were observed in the cytoplasm in a dispersing fashion (Figure 1f). It gave an orangish fluorescence in merged images (Figure 1f, fourth column). It suggested that G_4_‐SNAP‐FITC and Ce6 were in good location consistency. To further probe this phenomenon, the intensity of red and green fluorescence inside cells was profiled, respectively. Both curves matched well with each other along three random lines in enlarged confocal microscopy images (Figure S4). The good accordance of G_4_‐SNAP‐FITC and Ce6 indicated that most Ce6 was brought into cells with G_4_‐SNAP in composites form. As mentioned before, NO is a vulnerable molecule with a rather short half‐live and diffusion distance under physiological conditions. It usually requires releasing in close proximity of targets to obtain the best bioeffects. Endocytosis of G_4_‐SNAP/Ce6 composites rather than separated free components brings Ce6 within nanometers of NO‐releasing sites. It was beneficial to amplify the joint effects of NO and PDT.

### 3.3 GSH initiated NO release and intracellular delivery of Ce6

**FIGURE 2:**
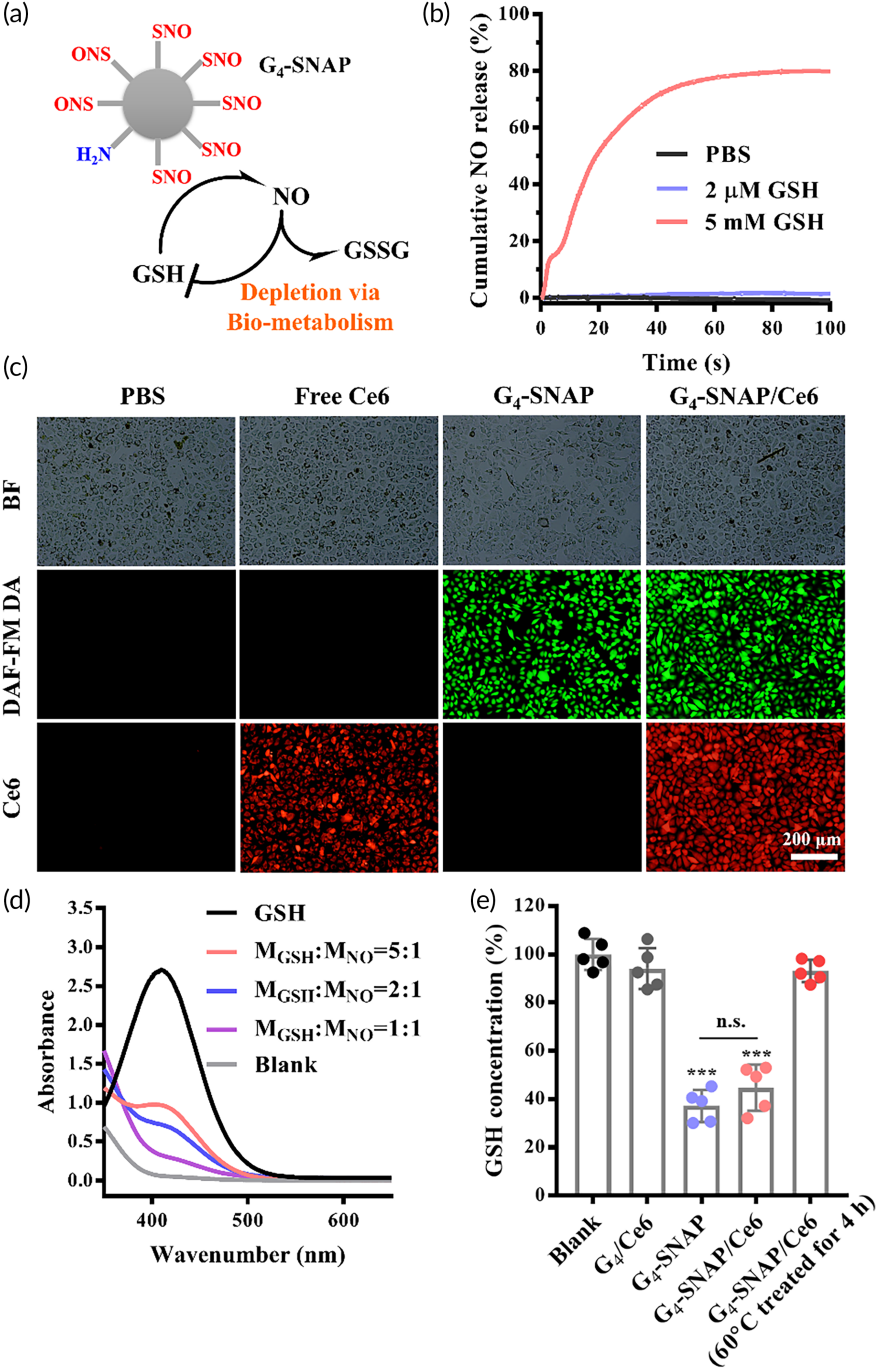
(a) Illustration of GSH depletion by G_4_‐SNAP and subsequent NO release. (b) Real‐time track profiles of NO release from G_4_‐SNAP with 0 μM, 2 μM, and 5 mM of GSH in PBS. (c) Fluorescent microscopic images of intracellular delivery of NO and Ce6. (d) UV–Vis spectra of remaining GSH after incubation with different ratios of G_4_‐SNAP (chromogen by Ellman agent) and (e) intracellular GSH level of A375 cells after different treatments (scale bar = 200 μm). Laser intensity: 0.2 W/cm^2^, 1 min. Ce6, chlorin e6; GSH, glutathione; NO, nitric oxide; PBS, phosphate‐buffered saline; SNAP, *S*‐nitroso‐*N*‐acrylate penicillamine

It is reported that the GSH concentration inside cells is thousands of times higher than that outside cells (5–10 mM vs. 2 μM). Such a huge difference is ideal as a trigger for specific NO release (Figure 2a). The GSH stimulated NO release from SNAP conjugated dendrimer was tracked in real‐time in PBS buffer (Figure 2b). When GSH was absent, barely any NO was detected (Figure 2b, black line). Two micromolar of GSH only gave rise to a moderate NO release (Figure 2b, purple line). However, when 5 mM of GSH was present, the NO‐releasing rate was dramatically accelerated to 60 times that of 2 μM GSH during the initial 20 s (Figure 2b, red line). After 80 s, 80% of NO was calculated to be released. The fast release of NO under the intracellular level of GSH was desirable to deliver most of NO into the targeted cells.

The NO release in A375 cells was then visualized using DAF‐FM DA as a probe. Bright green fluorescence was observed in cells treated with either G_4_‐SNAP or G_4_‐SNAP/Ce6 (Figure 2c, third and fourth columns), suggesting the efficient intracellular release of NO. On the other hand, cells treated with G_4_‐SNAP/Ce6 presented brighter red fluorescence of Ce6 than cells cultured with free Ce6 (Figure 2c, second and fourth columns), possibly due to the higher zeta potential, which promotes cellular uptake. Taking together, these data demonstrated that the dendrimer composites were a potent vehicle for intracellular codelivery of NO and Ce6.

### 3.4 Depletion of GSH by G_4_‐SNAP/Ce6

The GSH consumption by G_4_‐SNAP/Ce6 was critical in improving the efficiency of subsequent PDT. As determined by the Ellman assay using a standard curve method (Figure S5), the remaining GSH in PBS decreased constantly with the elevated ratio between RSNOs and GSH. When the molar concentrations of RSNOs and GSH were equal, about 90% of GSH was eliminated only after 30 min incubation (Figure 2d). The GSH concentration in cells also reduced dramatically to less than 50% after being cultured with either G_4_‐SNAP or G_4_‐SNAP/Ce6 kept in the dark for 3 h (Figure 2e). However, the GSH depleting ability was entirely lost, when G_4_‐SNAP/Ce6 was heated to 60°C for 4 h before cellular incubation to prematurely disrupt RSNOs and release NO (92%; Figure S6). The results demonstrated the necessity of G_4_‐SNAP in depleting GSH. It also proved G_4_‐SNAP as a favorable GSH scavenger.

### 3.5 Enhancement of ROS production and generation of RNS

**FIGURE 3:**
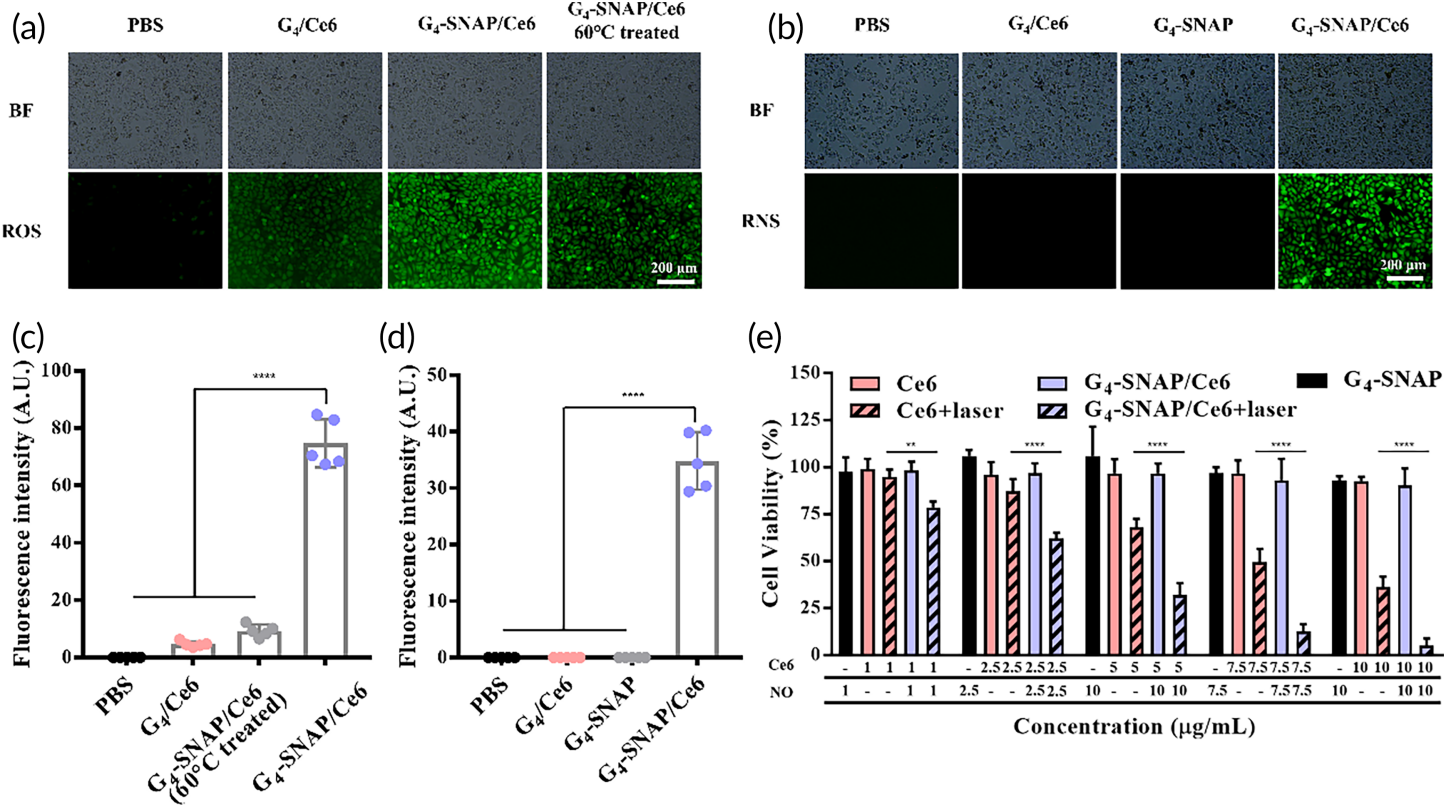
Fluorescent microscopic images of (a) ROS inside A375 cells visualized by DCFH‐DA probe and (b) RNS inside A375 cells visualized by DCF‐FM DA probe (scale bar = 200 μm); Relative fluorescent intensity of (c) ROS probe in cells (d) RNS probe in cells with different treatments and (e) cell viability after different treatments. Laser intensity: 0.2 W/cm^2^, 1 min. RNS, reactive nitrogen species; ROS, reactive oxygen species

ROS is the major element in PDT to kill cancer cells. Its abundance is vital for the therapeutic outcome. After exposure to a 660 nm laser for 1 min, green fluorescence emerged in cells treated with G_4_‐SNAP/Ce6, indicating the presence of a high level of ROS (Figure 3a, third column). However, the fluorescence inside cells was dimmer, when G_4_‐SNAP/Ce6 was preheated before cellular incubation (Figure 3a, fourth column and Figure 3c). G_4_‐SNAP/Ce6 would lose its ability to consume GSH after heat treatment, incapable of preventing GSH from scavenging ROS. It proved that G_4_‐SNAP could boost ROS generation of Ce6 via efficient GSH depletion. Moreover, RNS generated in G_4_‐SNAP/Ce6 treated cells were the most among all the groups after laser exposure (Figure 3b, third column and Figure 3d). It demonstrated the indispensability of both ROS and NO for the production of RNS. Such synergetic action between NO and Ce6 was helpful to strengthen the lethal effects of PDT.

### 3.6 Cell toxicity evaluation

The cell viability was then quantified using a standard CCK‐8 assay. Without laser irradiation, cells incubated with ether G_4_‐SNAP or G_4_‐SNAP/Ce6 demonstrated relatively high viability at all tested concentrations (Figure 3e). Low toxicity was required to minimize the side effects of G_4_‐SNAP/Ce6 composites. However, when exposed to 660 nm light, G_4_‐SNAP/Ce6 (10 μg/ml equivalent of SNAP and Ce6, predetermined by UV–vis spectrum according to standard curves) remarkably reduced the viability of A375 to 5%. It was almost 10 times lower than that of cells treated with the same amount of free Ce6 (10 μg/ml) (Figure 3e). The lethal cancer cell ablation was attributed to the ROS boost and RNS generation via the above‐mentioned GSH depletion and NO production. It is worth noting that the power of the laser was relatively low, and the time of light exposure was also short compared with other published work. The results proved the in vitro effectiveness of G_4_‐SNAP/Ce6 as an anticancer PDT agent.

### 3.7 Fabrication and characterization of G_4_‐SNAP/Ce6 embedded MNs

**FIGURE 4:**
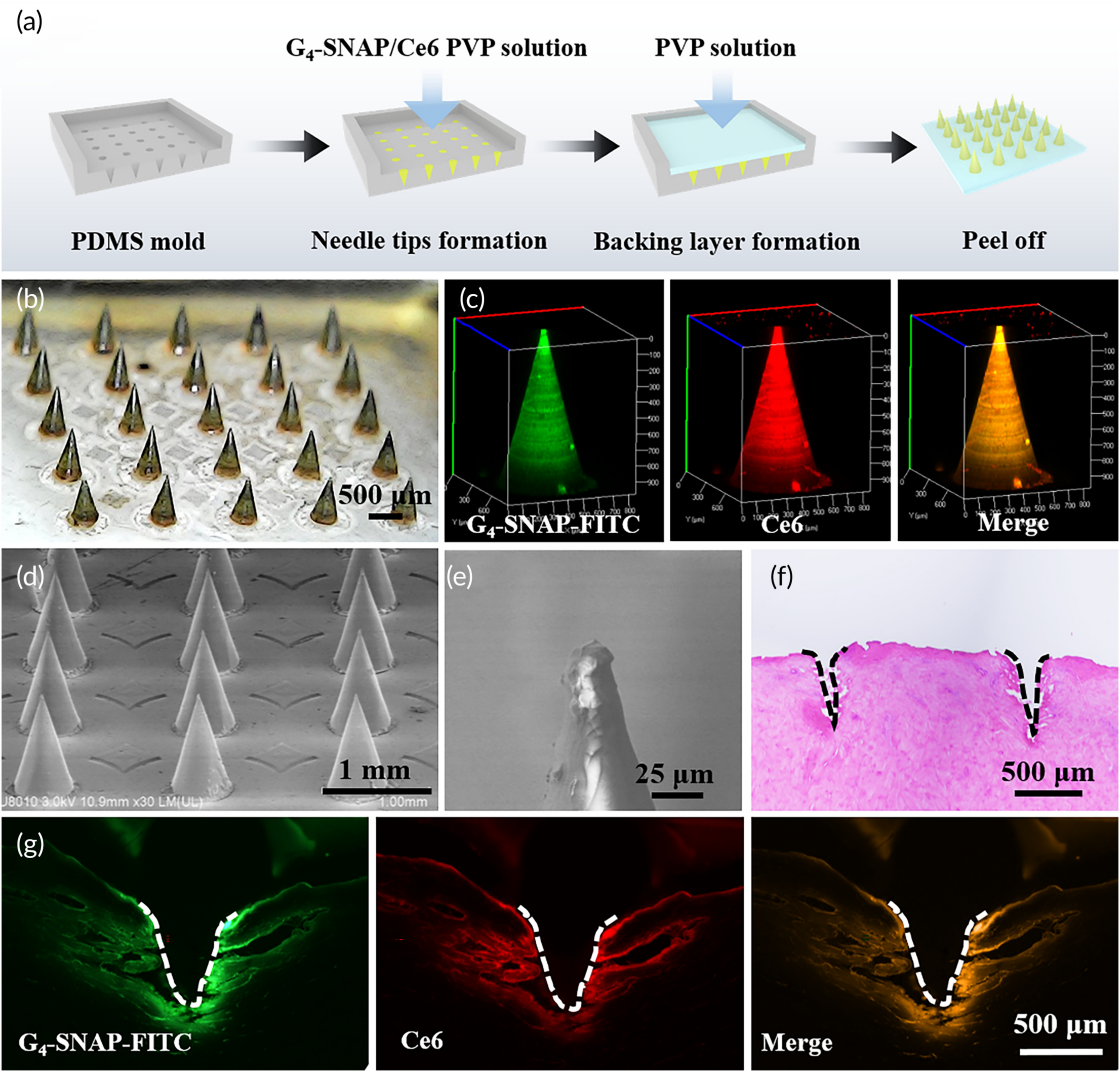
(a) G_4_‐SNAP/Ce6 loaded MNs fabricated using a two‐step casting method via PDMS molding. (b) Optical image of MNs patch. Scale bar = 500 μm. (c) 3D reconstruction images of G_4_‐SNAP‐FITC and Ce6 distribution in MNs. Scanning electron microscopy images of (d) MNs array and (e) needle tip. (f) Histology confirming MNs penetration in porcine skin. (g) Fluorescence images of G_4_‐SNAP‐FITC and Ce6 distribution in porcine skin after MNs application for 5 min. Scale bar = 500 μm. 3D, three‐dimensional; Ce6, chlorin e6; FITC, fluorescein isothiocyanate; MN, microneedle; PDMS, polydimethylsiloxane; SNAP, *S*‐nitroso‐*N*‐acrylate penicillamine

G_4_‐SNAP/Ce6 embedded MNs were fabricated by the molding method via a two‐step casting process (Figure 4a). These molds were replicated from male metal MNs molds (height: ~1000 μm, base width: ~500 μm, spacing: ~1000 μm, 5 × 5 array) by thermally initiated polymerization of PDMS prepolymer. PVP was employed as a polymeric matrix for MNs fabrication. G_4_‐SNAP/Ce6 composites were only loaded in MN tips (Figure 4b). To further investigate the drug distribution in MN tips, G_4_‐SNAP‐FITC/Ce6 composites were loaded in MNs and evaluated by a confocal microscope. The 3D reconstruction images confirmed the consistent distribution of G_4_‐SNAP‐FITC and Ce6, as the bright yellowish color was observed in merged images (Figure 4c). It indicated the stability of the composites during the fabrication processes. The specification of MNs was revealed by SEM images. Each conical needle has a base radius of 250 μm, a height of 1000 μm, and a tip radius of ∼10 μm (Figure 4d,e). Porcine skin was used for following penetration tests of MNs. Histology analysis validated the successful tissue penetration (Figure 4f). Upon manual insertion for 5 min, these MNs created cracks in the skin at a depth of 500–600 μm. Surprisingly, bright red and green fluorescence from G_4_‐SNAP‐FITC and Ce6 was observed in porcine tissues far from MNs inserting sites (Figure 4g). The drug delivery efficiency of different MNs application times was evaluated. Over 80% of encapsulated composites could be administered to the skin within 5 min (Figure S7). Considering the short administrating time, the release of G_4_‐SNAP/Ce6 composites from the MNs and its uptake by surrounding tissues was quite fast and efficient. Moreover, the yellow to orange color in the merged image (Figure 4g, third column) confirmed the consistent distribution of G_4_‐SNAP‐FITC and Ce6 even in the skin tissue, further demonstrating the composites' stability during diffusion.

### 3.8 In vivo antitumor evaluation of PDT

**FIGURE 5:**
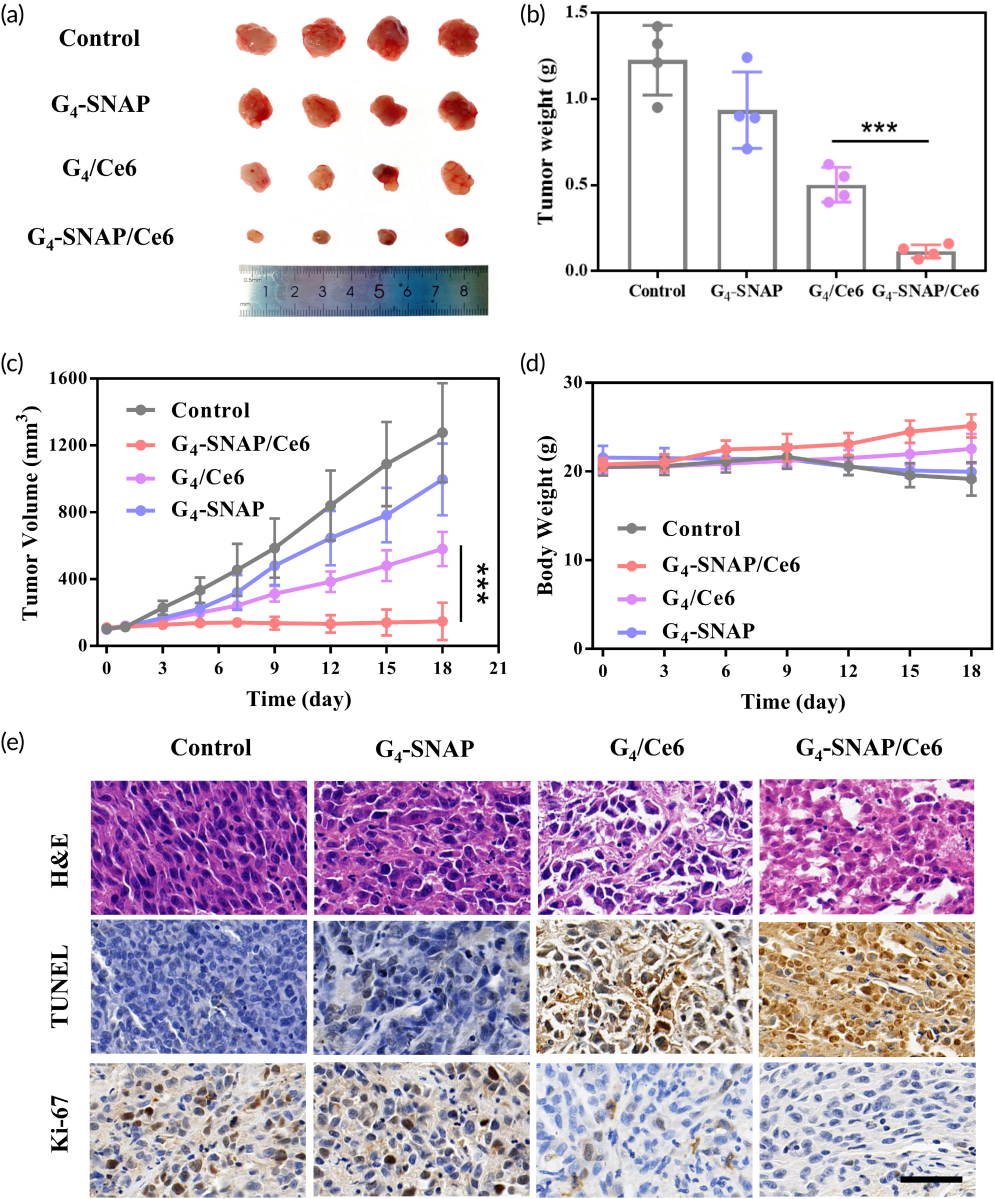
(a) Digital images and (b) weight summary of excised tumors 3 weeks after different treatments. (c) Changes in tumor volume with time. (d) Body weight changes of tumor‐bearing mice and (e) microscopic images of stained tumor slices with different treatments (scale bar = 100 μm). Laser intensity: 0.2 W/cm^2^, 1 min

Encouraged by promising results in vitro, we further evaluated the in vivo antitumor efficiency of G_4_‐SNAP/Ce6 embedded MNs. The MNs were administrated topically on tumors only once for 5 min and peeled off before further treatment. After 30 min, the tumor was irradiated by laser with relatively low power intensity and short exposure time (0.2 W/cm^2^, 1 min) to minimize collateral damage to surrounding tissues. Three weeks after therapy, tumors treated with G_4_‐SNAP/Ce6 MNs (25 μg Ce6 and 25 μg NO equivalent SNAP per patch) were the smallest among all groups (Figure 5a,b). The changes in tumor volume demonstrated that G_4_‐SNAP/Ce6 was the only treatment to halt tumor progression (Figure 5c). Meanwhile, G_4_‐SNAP (25 μg NO equivalent SNAP per patch) only slightly slowed the growth of the tumor. Even with G_4_/Ce6 (25 μg Ce6 per patch) treatment, the volume of tumors increased 5.5 times in 3 weeks, as a vivid illustration of the malignancy of melanoma tumors. Besides, the bodyweight of mice decreased obviously in groups treated with PBS or G_4_‐SNAP (Figure 5d). In comparison, mice treated with G_4_‐SNAP/Ce6 gained more weight than those with Ce6 during the 3 weeks. The results suggested that G_4_‐SNAP/Ce6 treatment was the most efficient in relieving the heavy burden of melanoma. Moreover, tumor slices were subjected to analysis to further access the anticancer effects of G_4_‐SNAP/Ce6. The composites demonstrated the most damage (H&E stain), the highest cell apoptosis (TUNEL stain), and the least proliferative cells (Ki‐67 stain) among all groups (Figure 5e). The significant difference in antitumor efficacy was due to the GSH‐scavenging effects and RNS generation of G_4_‐SNAP/Ce6, which boost the lethality of the combined therapy. It proved the excellent tumor suppression capacity of G_4_‐SNAP/Ce6‐loaded MNs in vivo.

Furthermore, the safety of the therapy was evaluated by a complete blood panel test and histological analyses of major organs and skin tissue (MN‐treated site). The blood test did not demonstrate noticeable changes in cell counts of red blood cells, white blood cells, blood platelet, lymphocyte, monocyte, and neutrophil, respectively (Figure S8). The results indicated that the therapy had desirable blood compatibility, nor did it promote significant immunological responses. Meanwhile, histological staining slices of major organs and skin tissues also presented negligible damages after different treatments (Figure S9). All these data proved the good safety of PDT mediated by G_4_‐SNAP/Ce6 MNs.

### 3.9 In vivo detection of GSH, RNS, and apoptotic markers

**FIGURE 6:**
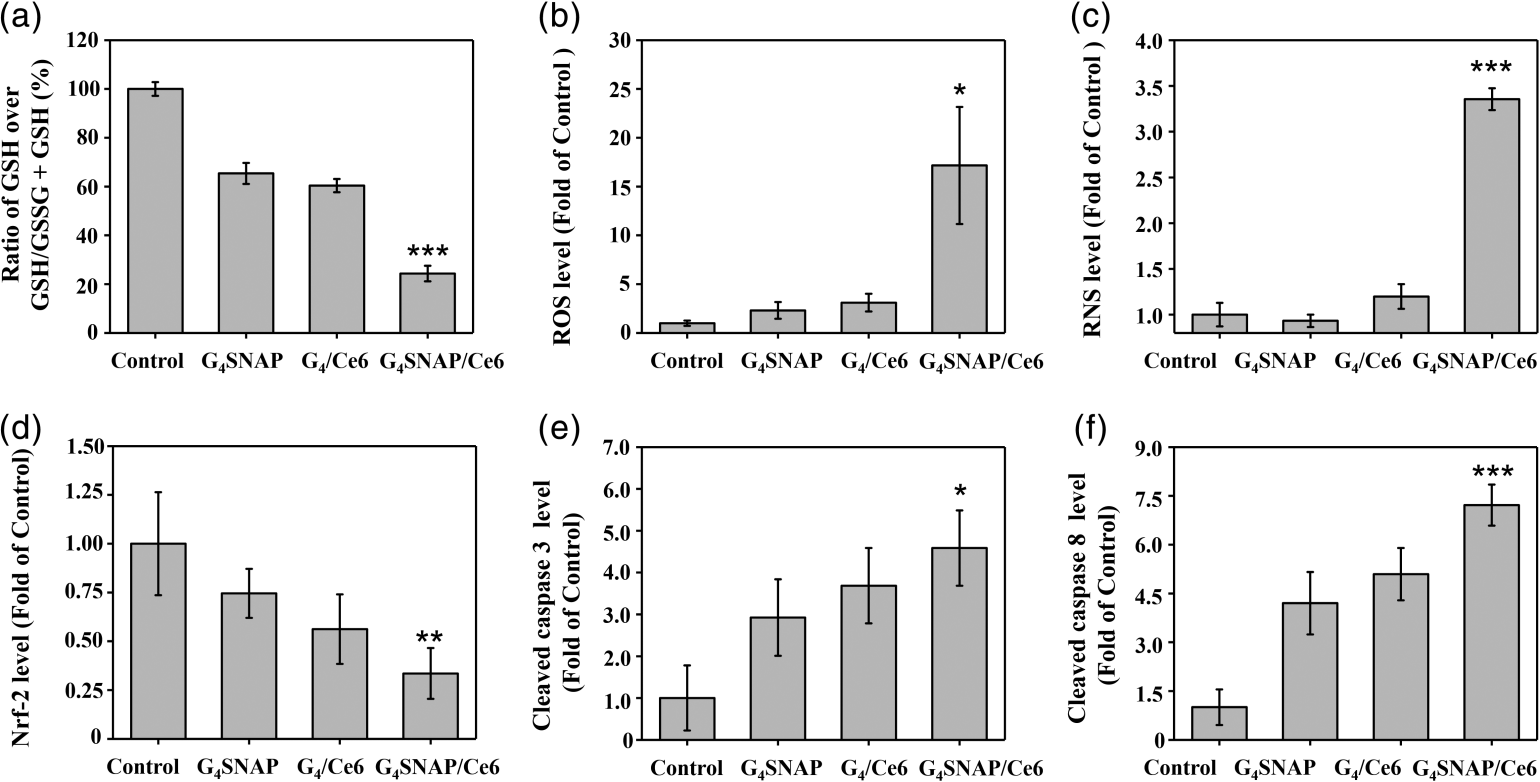
Relative level of (a) GSH, (b) ratio of GSH over total GSH/GSSG + GSH, (c) ROS, (d) RNS, (e) Nrf‐2, (f) TNF‐α, (g) cleaved caspase 3, and (h) cleaved caspase 8 in excised tumors 18 days after different treatments. GSH, glutathione; Nrf‐2, nuclear factor erythroid 2‐related factor 2; RNS, reactive nitrogen species; ROS, reactive oxygen species; TNF‐α, tumor necrosis factor‐α

The GSH concentration of tumors with different treatments was further quantified. As shown in Figure S10, G_4_‐SNAP treatment alone reduced the GSH level in tumors (36.4 ± 4.2% reduction compared with those in groups of blank MNs). Interestingly, the GSH depletion in G_4_/Ce6‐treated tumors demonstrated the scavenging effect of GSH on ROS (Figures 6a and S7). Therefore, ROS levels in tumors treated with G_4_/Ce6 alone only increased 3.1 ± 0.1 folds compared with those in groups of blank MNs (Figure 6b). However, tumor ROS levels in G_4_‐SNAP/Ce6 groups skyrocketed to 13.2 ± 4.0 folds relative to a blank (Figure 6b), with the least GSH level and the ratio of GSH/GSH + GSSG (Figures 6a and S7). Such results further confirmed that depletion of endogenous GSH was an effective way of boosting ROS generation in PDT. Meanwhile, only G_4_‐SNAP/Ce6 treatment generated sufficient RNS in the tumor (Figure 6c).

Furthermore, G_4_‐SNAP/Ce6 treatment significantly reduced the expression of Nrf‐2 (Figures 6d and S11). Nrf‐2 is closely related to the antioxidative stress activity of cells. The decrease in Nrf‐2 expression was assumed to sensitize the tumor to ROS. Accordingly, tumor tissue with G_4_‐SNAP/Ce6 treatment presented the highest level of cleaved caspase 3 and 8, suggesting a high level of apoptosis (Figure 6e,f). These results demonstrated that the boost of PDT via GSH depletion and RNS formation was the key factor for the preferrable antimelanoma effects of G_4_‐SNAP/Ce6‐loaded MNs.

## 4 CONCLUSION

In summary, MNs embedded with GSH‐scavenging G_4_‐SNAP and PDT agent Ce6 were successfully fabricated. The MNs can deliver encapsulated cargo in a fast and efficient manner to the subdermal tumor after topical administration. The released G_4_‐SNAP/Ce6 composites demonstrated remarkable GSH depleting ability. The generated NO further reacted with ROS from subsequent PDT, yielding more lethal RNS. The GSH consumption and RNS production significantly increased the anticancer efficacy of MNs mediated PDT on melanoma tumors. The tumor progression was halted for up to 18 days after treatment only once. The strategy enabled relatively low laser intensity and short exposure time with high precision during PDT, minimizing harmful side effects on surrounding healthy tissues. We believe that our study will provide new solutions to overcome the bottlenecks of PDT and enrich the tools to fight against skin tumors.

## AUTHOR CONTRIBUTIONS

**Fan Jia:** Conceptualization (lead); data curation (lead); investigation (lead); methodology (lead); validation (lead); writing – original draft (lead); writing – review and editing (lead). **Weijiang Yu:** Conceptualization (equal); data curation (lead); investigation (lead); methodology (lead); writing – original draft (lead). **Xinfang Li:** Formal analysis (equal); investigation (equal); methodology (equal); validation (equal). **Yonghang Chen:** Investigation (equal); methodology (equal); validation (equal). **Youxiang Wang:** Supervision (lead); project administration (lead); writing – review and editing (lead); funding acquisition(lead). **Jian Ji:** Project administration (lead); supervision (lead); writing – review and editing (lead); funding acquisition (lead).

## CONFLICT OF INTEREST

The authors declare no conflict of interest.

### PEER REVIEW

The peer review history for this article is available at https://publons.com/publon/10.1002/btm2.10352.

## Supporting information


**Appendix S1** Supplementary Information

## Data Availability

All data needed to evaluate the conclusion in this paper are demonstrated in the manuscript and/ or in the supporting information. Additional data related to this work may be requested from the authors.
