# Supplementary material for: Microneedles loaded with glutathione‐scavenging composites for nitric oxide enhanced photodynamic therapy of melanoma
Source: Bioeng Transl Med. 2022 Jun 17;8(1):e10352. doi: 10.1002/btm2.10352 (PMC9842046; doi:10.1002/btm2.10352)
Supplement: Supplementary file 1 — Appendix S1 Supplementary Information [file BTM2-8-e10352-s001.docx]

**Supporting Information**

**Microneedles loaded with Glutathione-scavenging Composites for Nitric Oxide Enhanced Photodynamic Therapy of Melanoma**

*Fan Jia^‡^, Weijiang Yu^‡^, Xinfang Li, Yonghang Chen, Youxiang Wang* and Jian Ji**

**Affiliations**

MOE Key Laboratory of Macromolecule Synthesis and Functionalization, Department of Polymer Science and Engineering, Zhejiang University, 310027, P. R. China.

*^‡^**These authors contributed equally to this work.*

**Co-corresponding author:* Dr. Youxiang Wang (yx_wang@zju.edu.cn), and Prof. Jian Ji (jijian@zju.edu.cn)


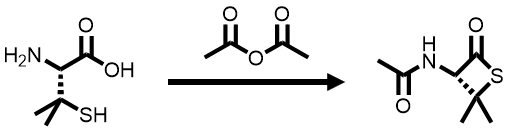


Figure S1. Synthesis procedure of N-acetylepenicillamine-S-thiolactone (NAP-thiolactone)


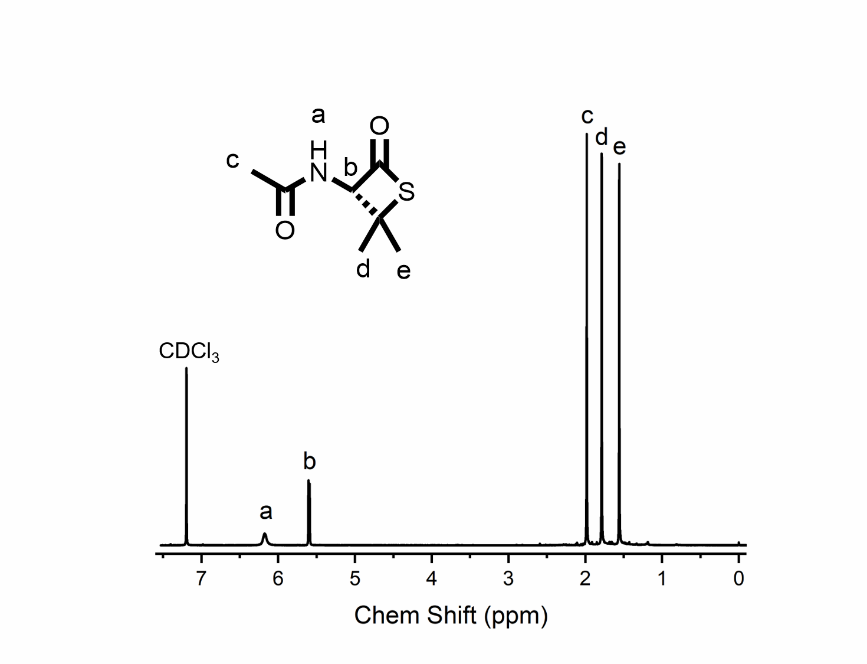


Figure S2. ^1^H NMR spectrum of NAP-thiolactone in CDCl_3_

Figure S3. Standard curve of Ce6 concentration and UV-Vis absorbance at 660 nm


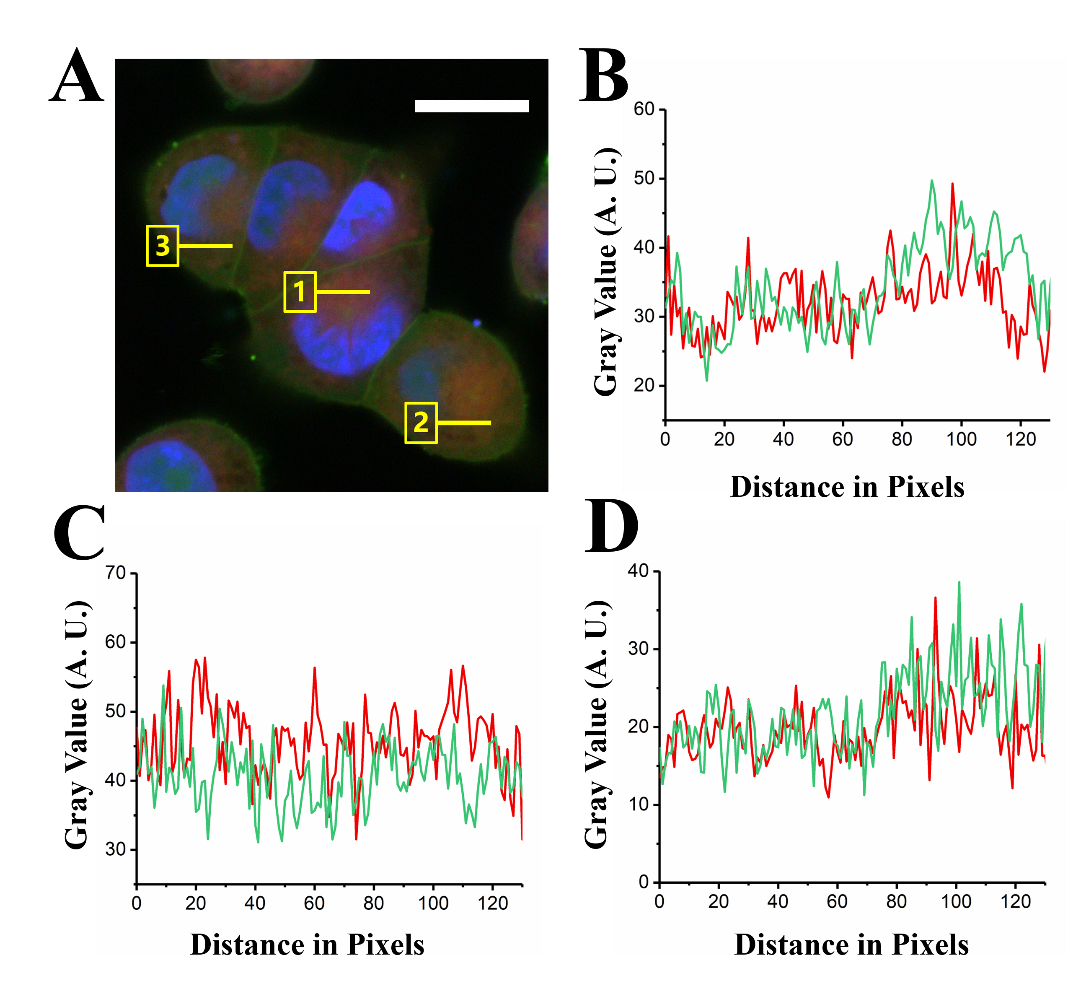


Figure S4. A) Confocal microscopic images of A-375 cells treated with FITC labeled G_4_-SNAP_55_ / Ce6 (scale bar: 40 μm)Zeta-potential of different dendrimers; B), C) and D) Fluorescent intensity profiles along yellow lines of 1, 2, and 3 in A, respectively (red curve: Ce6 and green curve: FITC)

Figure S5. Standard curve of GSH concentration and UV-Vis absorbance at 412 nm (chromogen by Ellman agent)

Figure S6. NO concentration after incubating at 60℃ for 4 h.

Figure S7. Drug delivery efficiency profile of G_4_-SNAP/Ce6 with different MNs application time.


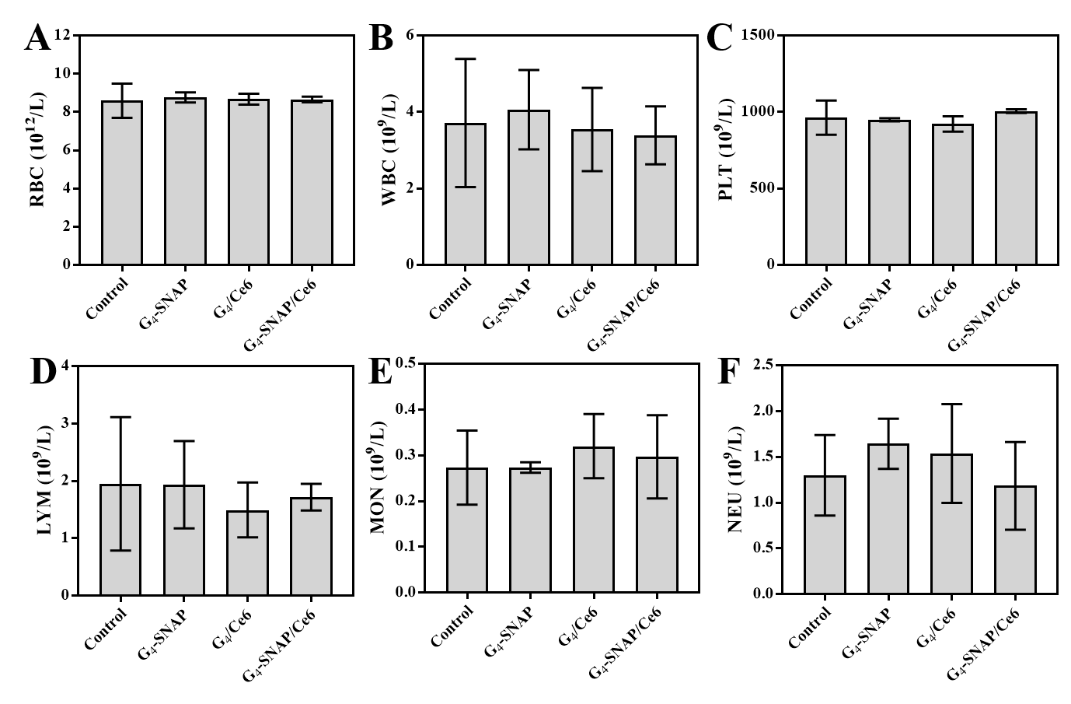


Figure S8. Complete blood panel tests of mice applied with G_4_-SNAP/Ce6 loaded MNs at day 7. The examined parameters include (A) red blood cells (RBC), (B) white blood cells (WBC), (C) blood platelet (PLT), (D) lymphocyte (LYM), (E) monocyte (MON), (F) neutrophil(NEU).


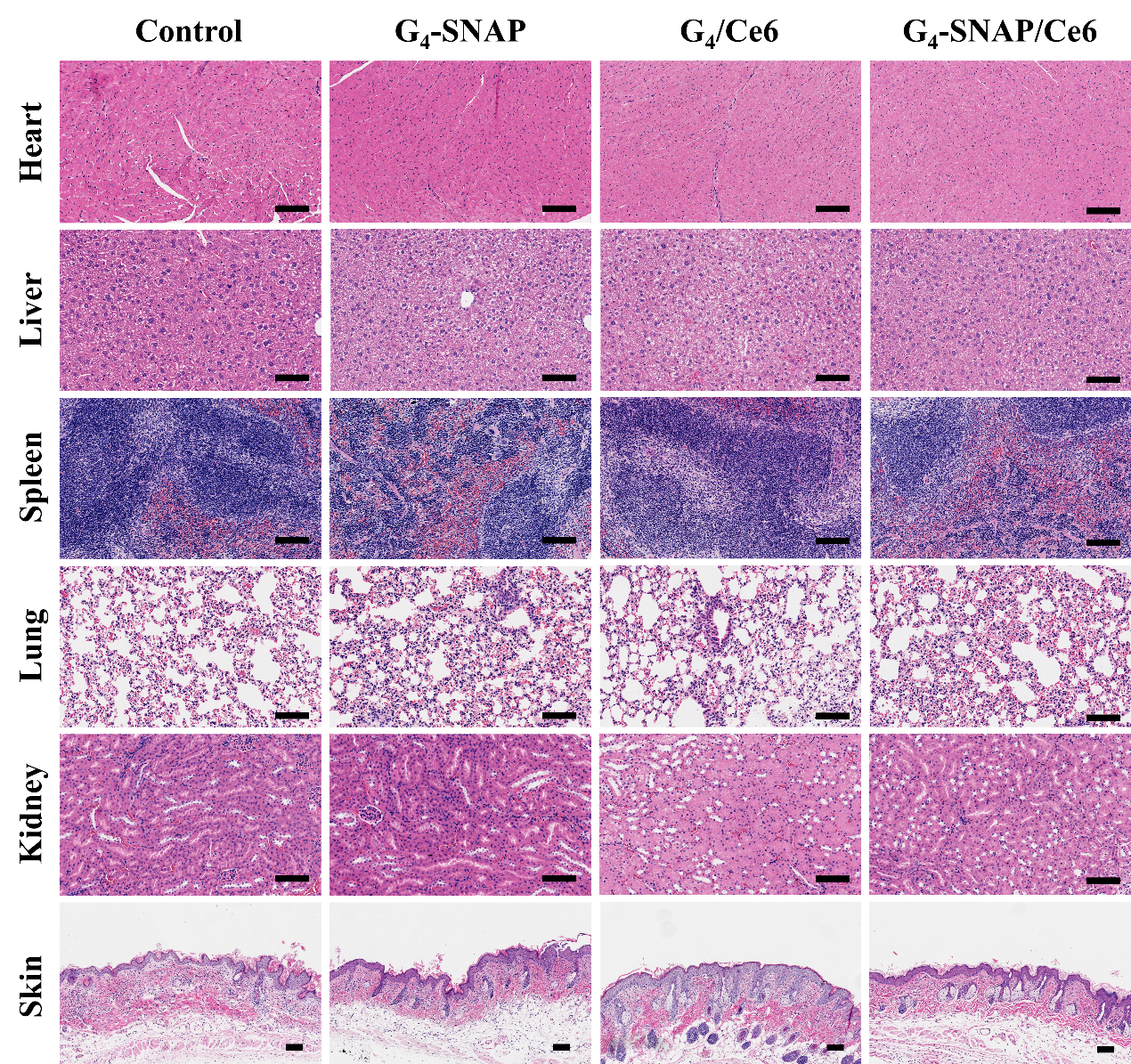


Figure S9. H&E-stained section of major organs and skin tissue (MN-treated site) from each group 7 d after treatment. Scale bar: 100 μm.


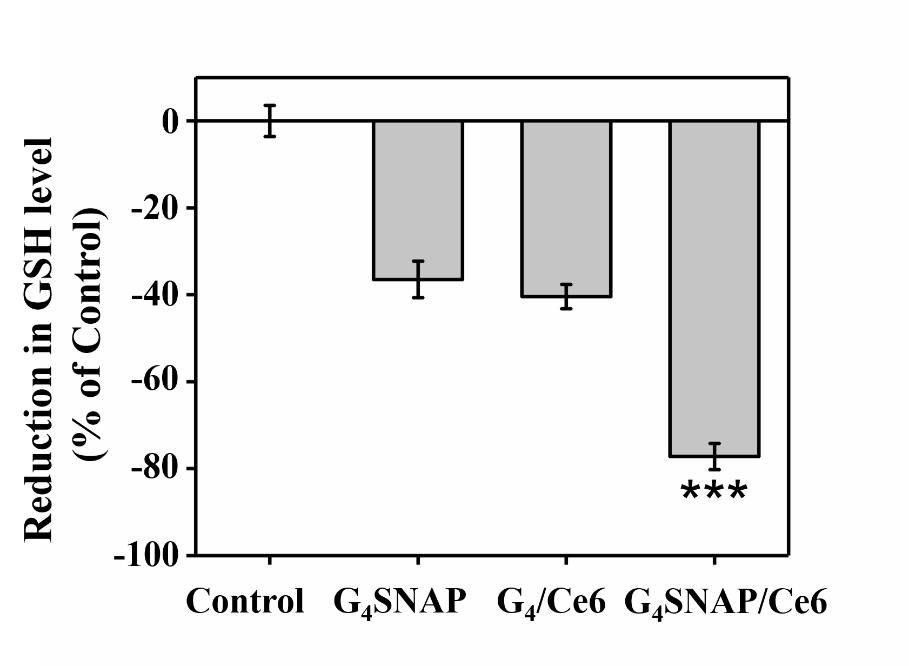


Figure S10. Relative level of GSH in excised tumors 3 weeks after different treatments.


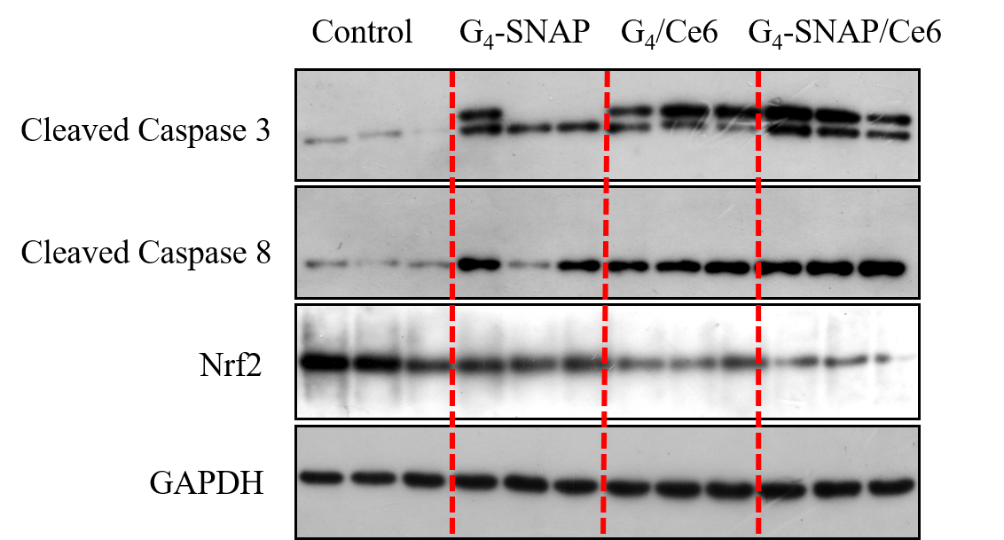


Figure S11. Relative expression of Nrf-2, Cleaved caspase 3 and Cleaved caspase 8

Figure S12. Standard curve of SNAP concentration and UV-Vis absorbance at 343 nm
